# Di-*tert*-butyl 2,2′-(biphenyl-2,2′-diyl­dioxy)diacetate

**DOI:** 10.1107/S1600536808019764

**Published:** 2008-07-05

**Authors:** Qamar Ali, Farooq Ibad, Muhammad Raza Shah, Donald VanDerveer

**Affiliations:** a HEJ Research Institute of Chemistry, International Center for Chemical and Biological Sciences, University of Karachi, Karachi 75270, Pakistan; b Chemistry Department, Clemson University, Clemson, SC 29634-0973, USA

## Abstract

The title compound, C_24_H_30_O_6_, does not exhibit π–π inter­actions due to the steric effect of the bulky *tert*-butyl groups present in the mol­ecule. The presence of these groups at the 2 and 2′ positions hinders the free motion of the benzene rings relative to each other, causing them to adopt an anti­periplanar arrangement. The benzene rings are twisted by just under 50.96 (17)° with respect to each other. The carbonyl groups within the mol­ecule are directed in different directions, one towards the biphenyl group and the other away from it. The mol­ecules are linked together by C=O⋯H—C hydrogen bonds.

## Related literature

For general background on chemical and biological studies of biphenyl compounds, see: Toshiaki *et al.* (2007 ▶); Kamoda *et al.* (2006 ▶); Makarov *et al.* (2005 ▶); Weisburger *et al.* (1967 ▶); Spivey *et al.* (1999 ▶); Sisson *et al.* (2006 ▶); Litvinchuk *et al.* (2004 ▶); Baudry *et al.* (2006 ▶). For the crystal structures of related compounds, see: Ali *et al.* (2008 ▶); Ibad *et al.* (2008 ▶).
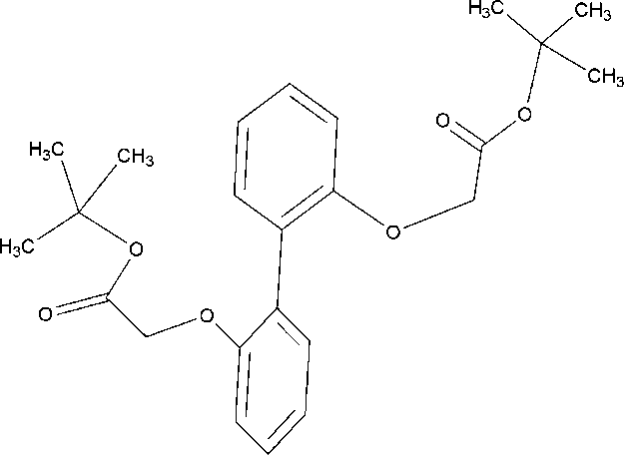


## Experimental

### 

#### Crystal data

C_24_H_30_O_6_
*M* *_r_* = 414.48
Triclinic, 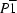

*a* = 7.7458 (15) Å
*b* = 12.112 (2) Å
*c* = 13.480 (3) Å
α = 67.36 (3)°
β = 82.11 (3)°
γ = 82.68 (3)°
*V* = 1152.3 (4) Å^3^
*Z* = 2
Mo *K*α radiation
μ = 0.09 mm^−1^
*T* = 153 (2) K
0.48 × 0.38 × 0.19 mm

#### Data collection

Rigaku Mercury CCD diffractometer
Absorption correction: multi-scan (*REQAB*; Jacobson, 1998 ▶) *T*_min_ = 0.960, *T*_max_ = 0.984
8742 measured reflections
4191 independent reflections
3687 reflections with *I* > 2σ(*I*)
*R*_int_ = 0.012

#### Refinement

*R*[*F* ^2^ > 2σ(*F* ^2^)] = 0.042
*wR*(*F* ^2^) = 0.113
*S* = 1.06
4191 reflections
271 parameters
H-atom parameters constrained
Δρ_max_ = 0.20 e Å^−3^
Δρ_min_ = −0.20 e Å^−3^

### 

Data collection: *CrystalClear* (Rigaku/MSC, 2006 ▶); cell refinement: *CrystalClear*; data reduction: *CrystalClear*; program(s) used to solve structure: *SHELXTL* (Sheldrick, 2008 ▶); program(s) used to refine structure: *SHELXTL*; molecular graphics: *SHELXTL*; software used to prepare material for publication: *SHELXTL*.

## Supplementary Material

Crystal structure: contains datablocks I, global. DOI: 10.1107/S1600536808019764/ez2127sup1.cif

Structure factors: contains datablocks I. DOI: 10.1107/S1600536808019764/ez2127Isup2.hkl

Additional supplementary materials:  crystallographic information; 3D view; checkCIF report

## Figures and Tables

**Table 1:** Hydrogen-bond geometry (Å, °)

*D*—H⋯*A*	*D*—H	H⋯*A*	*D*⋯*A*	*D*—H⋯*A*
C2—H2*A*⋯O2^i^	0.99	2.51	3.482 (2)	166
C20—H20*C*⋯O5^ii^	0.98	2.47	3.414 (2)	162

Symmetry codes: (i) 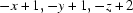; (ii) 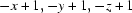.

**Fig. 1.**
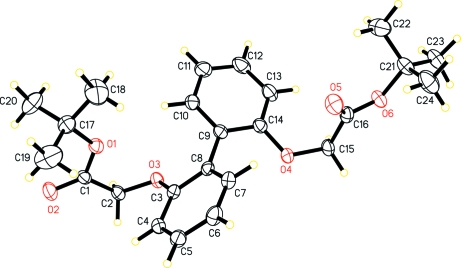
Crystal Structure of (I) showing the atom labelling scheme (50% ellipsoids).

**Fig. 2.**
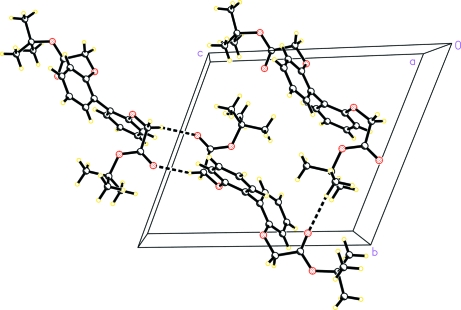
Partial packing diagram viewed along the a axis.

## supplementary crystallographic information

### Comment

Biphenyl moieties have been found to act as pharmacophores in many biological studies such as antimycobacterial testing (Kamoda *et al.*, 2006). Several derivatives of biphenyl are reported to be potential inhibitors of HRV-2 (Makarov *et al.*, 2005). However, they also show carcinogenic activity (Weisburger *et al.*, 1967). Furthermore, they occupy a unique place in various classes of organic compounds not only due to their prevalence as the core framework of numerous natural products, but also for their use as chiral reagents, as chiral phases for chromatography, and as chiral nucleophilic catalysts (Spivey *et al.*, 1999). Biphenyl derivatives are also used as precursors for the synthesis of oligo(*p*-phenylene)s (Sisson *et al.*, 2006). Oligo(*p*-phenylene)s have been extensively studied in the domain of artificial ion channels (Litvinchuk *et al.*, 2004). Our interest in the synthesis of biphenyl derivatives stems from the fact that we wish to attach macrocycles like porphyrins and calix[4]arenes to oligo(*p*-phenylene)s to obtain functionalized pores (Baudry *et al.*, 2006). In order to achieve these goals the synthesis of a number of biphenyl derivatives has been accomplished (Ali *et al.*, 2008; Ibad *et al.*, 2008). In this paper we report the synthesis and crystal structure of the title compound (I).

The OCH_2_C(═O)OC(CH_3_)_3_ residues are twisted away fom the biphenyl, as seen in the value of the C14—O4—C15—C16 torsion angle of 67.83 (14). The crystal packing diagram (Fig. 2) shows that there are fairly strong C—H···O interactions that are 0.2 Å less than the sum of the van der Waals radii, which results in the molecules forming chains in the c-direction.

### Experimental

K_2_CO_3_ (414 mg, 3 mmol) and 2,2'-dihydroxybiphenyl (186 mg, 1 mmol) in 15 ml of acetone were stirred for 10 minutes, followed by addition of tertiary butyl bromoacetate (371 mg, 3 mmol). The reaction mixture was stirred at room temperature for three hours. Solvent was evaporated under reduced pressure and the residue was dissolved in a mixture of water (50 ml) and dichloromethane (50 ml). The aqueous layer was extracted three times with dichloromethane.The combined organic phases were evaporated under reduced pressure and the solid residue was dissolved in hot hexane. Slow evaporation of hot hexane gave colorless crystals (736 mg) in 80% yield.

### Refinement

All H atoms were geometrically fixed and allowed to ride on the corresponding non-H atom with C—H = 0.96 Å and *U*_iso_(H) = 1.5*U*_eq_(C) of the attached C atom for methyl H atoms and 1.2*U*_eq_(C) for other H atoms.

### Crystal data

**Table:**

C_24_H_30_O_6_	*Z* = 2
*M**_r_* = 414.48	*F*_000_ = 444
Triclinic, *P*1	*D*_x_ = 1.195 Mg m^−^^3^
*a* = 7.7458 (15) Å	Mo *K*α radiation λ = 0.71073 Å
*b* = 12.112 (2) Å	Cell parameters from 3800 reflections
*c* = 13.480 (3) Å	θ = 3.0–26.4º
α = 67.36 (3)º	µ = 0.09 mm^−^^1^
β = 82.11 (3)º	*T* = 153 (2) K
γ = 82.68 (3)º	Chip, colorless
*V* = 1152.3 (4) Å^3^	0.48 × 0.38 × 0.19 mm

### Data collection

**Table:**

Rigaku Mercury CCD (2x2 bin mode) diffractometer	4191 independent reflections
Radiation source: Sealed Tube	3687 reflections with *I* > 2σ(*I*)
Monochromator: Graphite Monochromator	*R*_int_ = 0.012
Detector resolution: 14.6306 pixels mm^-1^	θ_max_ = 25.4º
*T* = 153(2) K	θ_min_ = 2.9º
ω scans	*h* = −9→9
Absorption correction: multi-scan(REQAB; Jacobson, 1998)	*k* = −14→14
*T*_min_ = 0.960, *T*_max_ = 0.984	*l* = −13→16
8742 measured reflections	

### Refinement

**Table:**

Refinement on *F*^2^	Secondary atom site location: difference Fourier map
Least-squares matrix: full	Hydrogen site location: inferred from neighbouring sites
*R*[*F*^2^ > 2σ(*F*^2^)] = 0.042	H-atom parameters constrained
*wR*(*F*^2^) = 0.113	*w* = 1/[σ^2^(*F*_o_^2^) + (0.0624*P*)^2^ + 0.3008*P*] where *P* = (*F*_o_^2^ + 2*F*_c_^2^)/3
*S* = 1.06	(Δ/σ)_max_ < 0.001
4191 reflections	Δρ_max_ = 0.20 e Å^−^^3^
271 parameters	Δρ_min_ = −0.20 e Å^−^^3^
Primary atom site location: structure-invariant direct methods	Extinction correction: none

### Special details

**Table:**

Geometry. All e.s.d.'s (except the e.s.d. in the dihedral angle between two l.s. planes) are estimated using the full covariance matrix. The cell e.s.d.'s are taken into account individually in the estimation of e.s.d.'s in distances, angles and torsion angles; correlations between e.s.d.'s in cell parameters are only used when they are defined by crystal symmetry. An approximate (isotropic) treatment of cell e.s.d.'s is used for estimating e.s.d.'s involving l.s. planes.
Refinement. Refinement of *F*^2^ against ALL reflections. The weighted *R*-factor *wR* and goodness of fit *S* are based on *F*^2^, conventional *R*-factors *R* are based on *F*, with *F* set to zero for negative *F*^2^. The threshold expression of *F*^2^ > σ(*F*^2^) is used only for calculating *R*-factors(gt) *etc*. and is not relevant to the choice of reflections for refinement. *R*-factors based on *F*^2^ are statistically about twice as large as those based on *F*, and *R*- factors based on ALL data will be even larger.

### Fractional atomic coordinates and isotropic or equivalent isotropic displacement parameters (Å^2^)

**Table:**

	*x*	*y*	*z*	*U*_iso_*/*U*_eq_	
O1	0.63964 (12)	0.48792 (8)	0.74773 (7)	0.0256 (2)	
O2	0.67544 (15)	0.42265 (9)	0.92572 (8)	0.0407 (3)	
O3	0.51537 (11)	0.70530 (8)	0.73639 (7)	0.0242 (2)	
O4	0.22493 (12)	0.95936 (8)	0.46008 (7)	0.0279 (2)	
O5	0.13650 (14)	0.94540 (8)	0.27408 (8)	0.0351 (2)	
O6	0.14967 (12)	1.14648 (8)	0.18911 (7)	0.0285 (2)	
C1	0.63803 (16)	0.49982 (12)	0.84152 (10)	0.0240 (3)	
C2	0.58898 (17)	0.62861 (11)	0.83262 (10)	0.0249 (3)	
H2A	0.5043	0.6279	0.8949	0.030*	
H2B	0.6951	0.6628	0.8381	0.030*	
C3	0.35750 (16)	0.67899 (11)	0.71761 (10)	0.0217 (3)	
C4	0.24448 (17)	0.60667 (11)	0.79977 (11)	0.0264 (3)	
H4A	0.2749	0.5724	0.8720	0.032*	
C5	0.08664 (17)	0.58520 (12)	0.77493 (12)	0.0307 (3)	
H5A	0.0099	0.5348	0.8302	0.037*	
C6	0.04097 (17)	0.63678 (12)	0.67037 (12)	0.0306 (3)	
H6A	−0.0676	0.6228	0.6539	0.037*	
C7	0.15448 (16)	0.70935 (11)	0.58920 (11)	0.0258 (3)	
H7A	0.1216	0.7451	0.5175	0.031*	
C8	0.31533 (15)	0.73087 (10)	0.61059 (10)	0.0211 (3)	
C9	0.44218 (16)	0.80053 (11)	0.52109 (10)	0.0219 (3)	
C10	0.61274 (17)	0.75052 (13)	0.50921 (11)	0.0285 (3)	
H10A	0.6473	0.6736	0.5598	0.034*	
C11	0.73297 (18)	0.81058 (15)	0.42515 (12)	0.0372 (4)	
H11A	0.8482	0.7749	0.4181	0.045*	
C12	0.68340 (19)	0.92246 (16)	0.35221 (12)	0.0403 (4)	
H12A	0.7657	0.9645	0.2952	0.048*	
C13	0.51479 (18)	0.97446 (14)	0.36101 (11)	0.0335 (3)	
H13A	0.4815	1.0514	0.3100	0.040*	
C14	0.39452 (16)	0.91327 (12)	0.44496 (10)	0.0245 (3)	
C15	0.16606 (19)	1.06562 (11)	0.37635 (11)	0.0284 (3)	
H15A	0.2491	1.1275	0.3596	0.034*	
H15B	0.0507	1.0966	0.4011	0.034*	
C16	0.15050 (16)	1.04284 (11)	0.27501 (11)	0.0255 (3)	
C17	0.67465 (19)	0.36897 (12)	0.73667 (12)	0.0309 (3)	
C18	0.6458 (3)	0.40082 (18)	0.61993 (16)	0.0636 (6)	
H18A	0.5237	0.4314	0.6089	0.095*	
H18B	0.6720	0.3291	0.6017	0.095*	
H18C	0.7230	0.4626	0.5734	0.095*	
C19	0.5431 (3)	0.28453 (16)	0.81140 (19)	0.0589 (5)	
H19A	0.5651	0.2644	0.8865	0.088*	
H19B	0.5544	0.2110	0.7960	0.088*	
H19C	0.4247	0.3235	0.8001	0.088*	
C20	0.8607 (2)	0.32095 (15)	0.75818 (16)	0.0461 (4)	
H20A	0.8746	0.3010	0.8345	0.069*	
H20B	0.9400	0.3820	0.7131	0.069*	
H20C	0.8886	0.2487	0.7410	0.069*	
C21	0.12658 (19)	1.15005 (13)	0.08041 (11)	0.0313 (3)	
C22	0.2762 (2)	1.07515 (15)	0.04516 (14)	0.0449 (4)	
H22A	0.3876	1.1053	0.0455	0.067*	
H22B	0.2633	1.0805	−0.0279	0.067*	
H22C	0.2744	0.9913	0.0951	0.067*	
C23	0.1343 (2)	1.28210 (13)	0.01004 (12)	0.0386 (4)	
H23A	0.2499	1.3070	0.0098	0.058*	
H23B	0.0444	1.3296	0.0387	0.058*	
H23C	0.1136	1.2950	−0.0639	0.058*	
C24	−0.0517 (2)	1.10931 (17)	0.08184 (13)	0.0458 (4)	
H24A	−0.1431	1.1605	0.1051	0.069*	
H24B	−0.0581	1.0258	0.1321	0.069*	
H24C	−0.0689	1.1153	0.0092	0.069*	

### Atomic displacement parameters (Å^2^)

**Table:**

	*U*^11^	*U*^22^	*U*^33^	*U*^12^	*U*^13^	*U*^23^
O1	0.0307 (5)	0.0248 (5)	0.0223 (5)	−0.0003 (4)	−0.0047 (4)	−0.0096 (4)
O2	0.0582 (7)	0.0344 (6)	0.0223 (5)	0.0101 (5)	−0.0090 (5)	−0.0052 (5)
O3	0.0245 (4)	0.0238 (4)	0.0226 (5)	−0.0039 (3)	−0.0061 (4)	−0.0049 (4)
O4	0.0285 (5)	0.0244 (5)	0.0218 (5)	0.0031 (4)	−0.0014 (4)	−0.0005 (4)
O5	0.0451 (6)	0.0234 (5)	0.0360 (6)	−0.0064 (4)	−0.0066 (4)	−0.0080 (4)
O6	0.0397 (5)	0.0235 (5)	0.0207 (5)	−0.0042 (4)	−0.0067 (4)	−0.0047 (4)
C1	0.0232 (6)	0.0285 (7)	0.0181 (6)	−0.0020 (5)	−0.0023 (5)	−0.0064 (5)
C2	0.0276 (6)	0.0280 (7)	0.0189 (6)	−0.0018 (5)	−0.0060 (5)	−0.0075 (5)
C3	0.0206 (6)	0.0192 (6)	0.0246 (6)	0.0003 (5)	−0.0034 (5)	−0.0077 (5)
C4	0.0255 (6)	0.0241 (6)	0.0239 (7)	0.0006 (5)	−0.0018 (5)	−0.0036 (5)
C5	0.0238 (6)	0.0252 (7)	0.0341 (8)	−0.0028 (5)	0.0012 (5)	−0.0024 (6)
C6	0.0214 (6)	0.0272 (7)	0.0407 (8)	−0.0036 (5)	−0.0055 (6)	−0.0088 (6)
C7	0.0248 (6)	0.0235 (6)	0.0281 (7)	0.0001 (5)	−0.0068 (5)	−0.0078 (5)
C8	0.0212 (6)	0.0173 (6)	0.0236 (6)	0.0008 (4)	−0.0023 (5)	−0.0071 (5)
C9	0.0229 (6)	0.0245 (6)	0.0196 (6)	−0.0039 (5)	−0.0037 (5)	−0.0085 (5)
C10	0.0243 (6)	0.0354 (7)	0.0244 (7)	0.0010 (5)	−0.0050 (5)	−0.0097 (6)
C11	0.0215 (6)	0.0561 (9)	0.0304 (7)	−0.0012 (6)	−0.0013 (5)	−0.0131 (7)
C12	0.0288 (7)	0.0581 (10)	0.0264 (7)	−0.0125 (7)	0.0020 (6)	−0.0061 (7)
C13	0.0331 (7)	0.0362 (8)	0.0245 (7)	−0.0080 (6)	−0.0037 (6)	−0.0021 (6)
C14	0.0250 (6)	0.0270 (6)	0.0215 (6)	−0.0027 (5)	−0.0040 (5)	−0.0082 (5)
C15	0.0355 (7)	0.0203 (6)	0.0234 (7)	0.0032 (5)	−0.0056 (5)	−0.0024 (5)
C16	0.0244 (6)	0.0211 (6)	0.0268 (7)	−0.0015 (5)	−0.0040 (5)	−0.0040 (5)
C17	0.0370 (7)	0.0250 (7)	0.0348 (8)	−0.0033 (6)	−0.0037 (6)	−0.0155 (6)
C18	0.1049 (17)	0.0525 (11)	0.0492 (11)	0.0072 (11)	−0.0259 (11)	−0.0343 (10)
C19	0.0529 (10)	0.0426 (9)	0.0859 (15)	−0.0215 (8)	0.0172 (10)	−0.0317 (10)
C20	0.0403 (9)	0.0373 (8)	0.0676 (12)	0.0044 (7)	−0.0056 (8)	−0.0292 (8)
C21	0.0412 (8)	0.0321 (7)	0.0215 (7)	−0.0085 (6)	−0.0057 (6)	−0.0083 (6)
C22	0.0565 (10)	0.0422 (9)	0.0359 (9)	−0.0074 (7)	0.0058 (7)	−0.0166 (7)
C23	0.0557 (9)	0.0333 (8)	0.0239 (7)	−0.0079 (7)	−0.0098 (6)	−0.0041 (6)
C24	0.0480 (9)	0.0551 (10)	0.0339 (8)	−0.0188 (8)	−0.0107 (7)	−0.0095 (8)

### Geometric parameters (Å, °)

**Table:**

O1—C1	1.3247 (16)	C12—H12A	0.9500
O1—C17	1.4921 (16)	C13—C14	1.3924 (19)
O2—C1	1.2044 (17)	C13—H13A	0.9500
O3—C3	1.3811 (15)	C15—C16	1.5168 (19)
O3—C2	1.4180 (16)	C15—H15A	0.9900
O4—C14	1.3805 (16)	C15—H15B	0.9900
O4—C15	1.4183 (16)	C17—C20	1.507 (2)
O5—C16	1.2038 (16)	C17—C18	1.511 (2)
O6—C16	1.3407 (17)	C17—C19	1.513 (2)
O6—C21	1.4839 (16)	C18—H18A	0.9800
C1—C2	1.5196 (18)	C18—H18B	0.9800
C2—H2A	0.9900	C18—H18C	0.9800
C2—H2B	0.9900	C19—H19A	0.9800
C3—C4	1.3939 (19)	C19—H19B	0.9800
C3—C8	1.4006 (18)	C19—H19C	0.9800
C4—C5	1.3910 (19)	C20—H20A	0.9800
C4—H4A	0.9500	C20—H20B	0.9800
C5—C6	1.380 (2)	C20—H20C	0.9800
C5—H5A	0.9500	C21—C23	1.517 (2)
C6—C7	1.391 (2)	C21—C22	1.518 (2)
C6—H6A	0.9500	C21—C24	1.520 (2)
C7—C8	1.3938 (17)	C22—H22A	0.9800
C7—H7A	0.9500	C22—H22B	0.9800
C8—C9	1.4950 (18)	C22—H22C	0.9800
C9—C10	1.3966 (19)	C23—H23A	0.9800
C9—C14	1.3967 (19)	C23—H23B	0.9800
C10—C11	1.389 (2)	C23—H23C	0.9800
C10—H10A	0.9500	C24—H24A	0.9800
C11—C12	1.377 (2)	C24—H24B	0.9800
C11—H11A	0.9500	C24—H24C	0.9800
C12—C13	1.386 (2)		
			
C1—O1—C17	122.25 (11)	C16—C15—H15B	109.3
C3—O3—C2	117.83 (10)	H15A—C15—H15B	108.0
C14—O4—C15	116.99 (11)	O5—C16—O6	125.86 (13)
C16—O6—C21	121.03 (10)	O5—C16—C15	123.97 (12)
O2—C1—O1	126.99 (13)	O6—C16—C15	110.14 (11)
O2—C1—C2	120.93 (12)	O1—C17—C20	109.60 (11)
O1—C1—C2	112.04 (11)	O1—C17—C18	102.02 (12)
O3—C2—C1	115.22 (10)	C20—C17—C18	111.65 (15)
O3—C2—H2A	108.5	O1—C17—C19	109.59 (12)
C1—C2—H2A	108.5	C20—C17—C19	112.56 (14)
O3—C2—H2B	108.5	C18—C17—C19	110.90 (16)
C1—C2—H2B	108.5	C17—C18—H18A	109.5
H2A—C2—H2B	107.5	C17—C18—H18B	109.5
O3—C3—C4	122.53 (11)	H18A—C18—H18B	109.5
O3—C3—C8	116.03 (11)	C17—C18—H18C	109.5
C4—C3—C8	121.43 (11)	H18A—C18—H18C	109.5
C5—C4—C3	119.31 (12)	H18B—C18—H18C	109.5
C5—C4—H4A	120.3	C17—C19—H19A	109.5
C3—C4—H4A	120.3	C17—C19—H19B	109.5
C6—C5—C4	120.34 (12)	H19A—C19—H19B	109.5
C6—C5—H5A	119.8	C17—C19—H19C	109.5
C4—C5—H5A	119.8	H19A—C19—H19C	109.5
C5—C6—C7	119.79 (12)	H19B—C19—H19C	109.5
C5—C6—H6A	120.1	C17—C20—H20A	109.5
C7—C6—H6A	120.1	C17—C20—H20B	109.5
C6—C7—C8	121.50 (12)	H20A—C20—H20B	109.5
C6—C7—H7A	119.2	C17—C20—H20C	109.5
C8—C7—H7A	119.2	H20A—C20—H20C	109.5
C7—C8—C3	117.62 (12)	H20B—C20—H20C	109.5
C7—C8—C9	120.83 (11)	O6—C21—C23	102.98 (11)
C3—C8—C9	121.45 (11)	O6—C21—C22	109.69 (12)
C10—C9—C14	117.97 (12)	C23—C21—C22	110.79 (13)
C10—C9—C8	119.72 (11)	O6—C21—C24	109.62 (12)
C14—C9—C8	122.25 (11)	C23—C21—C24	110.38 (13)
C11—C10—C9	121.56 (13)	C22—C21—C24	112.92 (13)
C11—C10—H10A	119.2	C21—C22—H22A	109.5
C9—C10—H10A	119.2	C21—C22—H22B	109.5
C12—C11—C10	119.28 (14)	H22A—C22—H22B	109.5
C12—C11—H11A	120.4	C21—C22—H22C	109.5
C10—C11—H11A	120.4	H22A—C22—H22C	109.5
C11—C12—C13	120.73 (13)	H22B—C22—H22C	109.5
C11—C12—H12A	119.6	C21—C23—H23A	109.5
C13—C12—H12A	119.6	C21—C23—H23B	109.5
C12—C13—C14	119.66 (13)	H23A—C23—H23B	109.5
C12—C13—H13A	120.2	C21—C23—H23C	109.5
C14—C13—H13A	120.2	H23A—C23—H23C	109.5
O4—C14—C13	123.23 (12)	H23B—C23—H23C	109.5
O4—C14—C9	115.98 (11)	C21—C24—H24A	109.5
C13—C14—C9	120.78 (12)	C21—C24—H24B	109.5
O4—C15—C16	111.40 (11)	H24A—C24—H24B	109.5
O4—C15—H15A	109.3	C21—C24—H24C	109.5
C16—C15—H15A	109.3	H24A—C24—H24C	109.5
O4—C15—H15B	109.3	H24B—C24—H24C	109.5
			
C17—O1—C1—O2	5.8 (2)	C8—C9—C10—C11	177.90 (12)
C17—O1—C1—C2	−176.69 (10)	C9—C10—C11—C12	0.5 (2)
C3—O3—C2—C1	63.42 (14)	C10—C11—C12—C13	−1.0 (2)
O2—C1—C2—O3	−168.21 (12)	C11—C12—C13—C14	0.4 (2)
O1—C1—C2—O3	14.12 (15)	C15—O4—C14—C13	9.65 (18)
C2—O3—C3—C4	20.01 (17)	C15—O4—C14—C9	−171.68 (11)
C2—O3—C3—C8	−160.90 (11)	C12—C13—C14—O4	179.23 (13)
O3—C3—C4—C5	179.27 (12)	C12—C13—C14—C9	0.6 (2)
C8—C3—C4—C5	0.22 (19)	C10—C9—C14—O4	−179.80 (11)
C3—C4—C5—C6	−1.2 (2)	C8—C9—C14—O4	2.91 (17)
C4—C5—C6—C7	0.8 (2)	C10—C9—C14—C13	−1.10 (19)
C5—C6—C7—C8	0.5 (2)	C8—C9—C14—C13	−178.39 (12)
C6—C7—C8—C3	−1.45 (19)	C14—O4—C15—C16	67.83 (14)
C6—C7—C8—C9	174.84 (12)	C21—O6—C16—O5	1.3 (2)
O3—C3—C8—C7	−178.03 (11)	C21—O6—C16—C15	−176.87 (11)
C4—C3—C8—C7	1.08 (18)	O4—C15—C16—O5	21.25 (18)
O3—C3—C8—C9	5.71 (17)	O4—C15—C16—O6	−160.51 (11)
C4—C3—C8—C9	−175.19 (11)	C1—O1—C17—C20	−66.57 (16)
C7—C8—C9—C10	−125.19 (13)	C1—O1—C17—C18	174.98 (14)
C3—C8—C9—C10	50.96 (17)	C1—O1—C17—C19	57.41 (17)
C7—C8—C9—C14	52.05 (17)	C16—O6—C21—C23	−179.93 (12)
C3—C8—C9—C14	−131.80 (13)	C16—O6—C21—C22	−61.94 (16)
C14—C9—C10—C11	0.54 (19)	C16—O6—C21—C24	62.58 (17)

### Hydrogen-bond geometry (Å, °)

**Table:**

*D*—H···*A*	*D*—H	H···*A*	*D*···*A*	*D*—H···*A*
C2—H2A···O2^i^	0.99	2.51	3.482 (2)	166
C20—H20C···O5^ii^	0.98	2.47	3.414 (2)	162

Symmetry codes: (i) −*x*+1, −*y*+1, −*z*+2; (ii) −*x*+1, −*y*+1, −*z*+1.
